# Royal Free Hospital

**Published:** 1852-11

**Authors:** 


ROYAL FREE HOSPITAL. *Physicians*—Drs. Chambers and Brinton. *Surgeons*—Messrs. Marsden, Gray, and T. Wakley. *Assistant Surgeon*—Mr. T. W. Cooke. *Medical Officer for Diseases of Children*—Mr. T. C. Jackson. *Dentist*—Mr. Robinson.

ST. THOMAS' HOSPITAL. *Consulting Physician*—Dr. Roots. *Physicians*—Drs. Barker, Leeson, and Risdon Bennett. *Assistant Physicians*—Drs. Goolden, Cohen, and Peacock. *Surgeons*—Messrs. Green,

South, and Mackmurdo. *Assistant Surgeons*—Messrs. Solly and Clark. *Midwifery Department. Physicians*—Drs. Waller and Griffith. *Ophthalmic Department. Surgeon*—Mr. Mackmurdo.

MEDICAL AND SURGICAL COLLEGE.—Anatomy Demonstrations, Messrs. Rainey and Crosby. Physiology and General Anatomy, Mr. Grainger. The Teeth, their Structure and Diseases, Mr. Saunders. Microscopical Anatomy, Messrs. Grainger and Rainey. Surgery, Messrs. Green and South. Post Mortem Examinations, Messrs. Adams and Bristowe. Descriptive and Surgical Anatomy, Mr. Clark. Theory and Practice of Medicine, Dr. Barker. Chemistry, Dr. Leeson. Ophthalmic Surgery, Mr. Mackmurdo. *Summer Lectures.*—Comparative Anatomy and Natural History, Dr. Meryon. Medical Jurisprudence, Dr. Barker. Materia Medica, Dr. Risdon Bennett. Midwifery, Dr. Waller. Botany, Mr. Bristowe. Pathology, Mr. Simon. Practical Chemistry, Dr. Gladstone.

